# Continuous palliative sedation until death: a qualitative study of palliative care clinicians’ experiences

**DOI:** 10.1186/s12904-024-01426-2

**Published:** 2024-04-18

**Authors:** Alexandra Guité-Verret, Jessica Boivin, Andrew M. R. Hanna, James Downar, Shirley H. Bush, Isabelle Marcoux, Diane Guay, Diane Tapp, Julie Lapenskie, Bruno Gagnon

**Affiliations:** 1 Department of Psychology, Université du Québec à Montréal, Montréal, Canada; 2 Réseau Québécois de Recherche en Soins Palliatifs et de fin de vie, Québec, Canada; 3 Department of Social and Preventive Medicine, Faculty of Medicine, Université Laval, Québec, Canada; 4 CHU Québec-Université Laval Research Centre, Québec, Canada; 5 Bruyère Research Institute, Ottawa, Canada; 6 Department of Medicine, University of Ottawa, Ottawa, Canada; 7 Ottawa Hospital Research Institute, University of Ottawa, Ottawa, Canada; 8 Faculty of Health Sciences, University of Ottawa, Ottawa, Canada; 9 Faculty of Medicine and Health Sciences, Université de Sherbrooke, Québec, Canada; 10 Faculty of Nursing, Université Laval, Québec, Canada; 11 Department of Family and Emergency Medicine, Université Laval, Québec, Canada

**Keywords:** Continuous palliative sedation, Deep sedation, Palliative care, End-of-life care, Assisted dying, Medical assistance in dying, Euthanasia, Qualitative

## Abstract

**Background:**

The practice of continuous palliative sedation until death is the subject of much medical and ethical debate, which is reflected in the inconsistency that persists in the literature regarding the definition and indications of palliative sedation.

**Aim:**

This study aims to gain a better understanding of palliative care clinicians’ experiences with continuous palliative sedation.

**Design:**

We conducted a qualitative study based on focus group discussions.

**Setting/participants:**

We conducted six focus groups with a total of 28 palliative care clinicians (i.e., 15 nurses, 12 physicians, and 1 end-of-life doula) from diverse care settings across Canada, where assisted dying has recently been legalized.

**Results:**

An interpretative phenomenological analysis was used to consolidate the data into six key themes: responding to suffering; grappling with uncertainty; adapting care to ensure ongoing quality; grounding clinical practice in ethics; combining medical expertise, relational tact, and reflexivity; and offering an alternative to assisted death.

**Conclusions:**

Interaction with the patient’s family, uncertainty about the patient’s prognosis, the concurrent practice of assisted dying, and the treatment of existential suffering influence the quality of sedation and indicate a lack of clear palliative care guidelines. Nevertheless, clinicians exhibit a reflective and adaptive capacity that can facilitate good practice.

## Background

Focused on accompanying dying individuals and their families, palliative care aims to relieve the suffering of patients at a physical, psychological, social, and existential level. When patients continue to experience severe uncontrollable symptoms despite optimal palliative care, continuous sedation, i.e., the reduction of the patient’s consciousness through sedatives until death, becomes a last-resort option [1]. Important discrepancies in practicing continuous sedation are reported in the literature, leading to a varying prevalence worldwide and a lack of a consensus on its definition [1–3]. Validated instruments exist for assessing sedation effects in palliative care, but recent systematic reviews highlight the need for clearer guidelines on identifying patients who may benefit from continuous sedation [4–6]. In addition, guideline recommendations are not always aligned with the reality of palliative care practice. [1, 7, 8] This dissonance is particularly evident in home care settings, where the applicability of guidelines is called into question. [9, 10] It is crucial to explicitly define and critically examine palliative sedation, including its potential risks and ethical considerations, to ensure the quality of palliative care [11]. 

The most common indications for continuous sedation are agitated delirium, pain and dyspnea, [4, 12, 13] while its use for existential suffering is controversial [14–17]. The indications for continuous sedation are linked to clinicians’ intention to offer a “good” death for the patient and their family [18–22]. Palliative care clinicians also encounter challenges in providing quality sedation, including fear that continuous sedation hastens death, conflicting wishes between patients and families, and disagreements within care teams [13, 23–25]. 

Another aspect that arises from the literature is the evolving nature of continuous sedation in light of the legalization of assisted death, also known as “medical assistance in dying” in Canada. In Western Europe and North America, assisted death has impacted continuous sedation practice, particularly regarding patients’ requests, clinicians’ intentions, and decision-making [26]. Patients and families tend to be more familiar with assisted death than continuous sedation, [26, 27] reflecting the limited public understanding of palliative care [28–30]. 

The aforementioned are primarily based on literature reviews and studies conducted in the past decade. There is a need for new empirical data that can provide insights into the quality of palliative sedation from the perspective of palliative care clinicians [4]. It is important to study what they consider to be the recent challenges they face in their practice.

## Methods

### Aim

The aim of this study was (1) to describe the experiences of clinicians from various palliative care settings in using continuous sedation, and (2) to explore how assisted death influences palliative sedation practices. This study is part of a wider research project aimed at developing a palliative care educational module for healthcare professionals.

### Methodological approach and position

This qualitative study followed the approach and guidelines of Interpretative Phenomenological Analysis [31], with theoretical underpinnings from phenomenology and hermeneutics. Interpretative Phenomenological Analysis aims to interpret participants’ personal experiences. [31, 32] It is based on a constructivist methodological position [33]. This position shapes our ontological and epistemological stance, assuming that reality is constructed by the context, including participants’ lived experiences, the social environment, and the interaction between participants and researchers [33]. We chose Interpretative Phenomenological Analysis for our research because it enabled us to understand the meaning of palliative care clinicians’ work and their ways of caring according to current practices and social context.

To ensure rigor of this study, we adhered to Tracy’s quality criteria in qualitative research [34] (see Table 1).

**Table 1:** Tracy’s eight criteria of quality in qualitative research

Criteria for quality	Means and practices through which criteria were achieved
Worthy topic	• Relevance with regard to societal events and priorities (e.g., lack of consensual definition on palliative sedation, legalization of assisted death) • In line with recent literature on palliative sedation
Rich rigor	• In-depth focus group discussions • Abundant and complex data • Appropriate theoretical constructs • Reflexive notes taken after each focus groups • Transparency about data collection and data analysis • Peer-discussions to deepen data analysis
Sincerity	• Reflexivity about preconceptions about palliative sedation and assisted death • Reflexivity about the researchers’ credentials, leading to collaboration and peer discussions to reflect on learning and practice • Transparency about methodological and theoretical assumptions • Recognition of the study limitations
Credibility	• “Thick” descriptions (e.g., rich descriptions in line with data complexity, numerous quotes from participants) • Immersion in the data to ascertain tacit knowledge
Resonance	• Evocative quotes leading to empathic resonance • Transferable findings
Significant contribution	• Practical and heuristic significance of the study (e.g., extending phenomenological knowledge on palliative sedation in the context of various settings and in the context of legalized assisted death) • Can lead to improve clinical practices
Ethics	• Ethics committee approval • Relational ethics (e.g., consideration of interdependence between researchers and participants, from data collection to data analysis)
Meaningful coherence	• Questions, paradigm, method and analysis in line with Interpretative Phenomenological Analysis (IPA) • Interconnections between aims, literature, data and interpretations

#### Participants and recruitment

Participants comprised 28 palliative care clinicians, including 15 nurses, 12 physicians, and 1 end-of-life doula, working in various care settings across Canada (see Table 2). We recruited participants via four palliative care organizations and snowball sampling and contacted them by e-mail. Eligibility criteria were: (a) be a healthcare professional; (b) provide continuous palliative sedation or have been involved in the care of at least one adult patient who received continuous palliative sedation when death was imminent; (c) work in a palliative care setting in Canada. No exclusion criteria were identified. In line with qualitative and phenomenological research recommendations [31], we recruited a small sample to describe clinicians’ lived experience in their specific care settings. We aimed for a total of 25 to 30 participants, forming four to six focus groups of four to eight individuals each. Recruitment ceased when the predetermined number of participants was reached.

**Table 2:** Sociodemographic data for the focus group participants

Characteristics	N (%)
**Age (years)**	
30–39	6 (21.4)
40–49	11 (39.3)
50–59	6 (21.4)
60–69	3 (10.7)
N/A	2 (7.1)
**Gender**	
Female	24 (85.7)
**Profession**	
Physician	12 (42.9)
Nurse	15 (53.6)
End-of-life doula	1 (3.6)
**Province**	
Quebec	7 (25.0)
Ontario	11 (39.3)
Alberta	3 (10.7)
British Columbia	6 (21.4)
New Brunswick	1 (3.6)
**Practice settings (all that apply)**	
Inpatient hospice	11 (23.9)
Community	19 (41.3)
Inpatient care	12 (26.1)
Long-term care	2 (4.3)
Retirement home	2 (4.3)
**Experience in palliative care (years)**	
< 5	4 (14.3)
5–10	10 (35.7)
11–20	6 (21.4)
21–30	5 (17.9)
> 30	1 (3.6)
N/A	2 (7.1)

This study was approved by the Research Ethics Committee of Centre Hospitalier Universitaire de Québec-Université Laval, where the study was conducted (no. 2023–6462, approved on July 9, 2022). All study participants provided written informed consent for study participation, data analysis, and publication.

### Data collection

Participants were invited to partake in one focus group to discuss their experiences of continuous palliative sedation. A focus group design was used to explore people’s knowledge and experiences, as it creates a space to reflect and share multiple views [35]. Group interactions are an integral part of this method, as they facilitate participants in clarifying their perceptions and understanding of the topic. [35, 36] A semi-structured interview guide was developed for this study by our research team for use in the focus groups.

Six semi-structured qualitative focus groups were conducted virtually using an online platform from October 2022 to March 2023. Each participant took part in only one of the six focus groups. Each focus group was conducted in either English (*n* = 5) or French (*n* = 1), by grouping participants according to their language. Focus groups consisted of 4 to 8 participants, with discussions lasting from 68 to 124 min. The interviewer started with the focus group with the following statement so that participants have a common definition to discuss: “We are referring only to continuous palliative sedation when death is imminent. Intermittent sedation and withdrawal or withholding or life-sustaining treatments are not intended topics of discussion for our focus group”. Through a series of open-ended questions, the interviewer elicited information on how the participants define continuous palliative sedation, their experiences, and any impact of assisted death and the COVID-19 pandemic on their practice (see Table 3). Focus groups were video recorded and transcribed verbatim.

**Table 3:** Interview guide for focus groups

Theme	Questions
Experiences and perception of palliative continuous sedation	According to your perception, how would you understand the concept of palliative continuous sedation? Can you tell me about your experience of continuous sedation for agitated delirium/dyspnea/psychological distress/pain/vomiting, etc.?
Situations when palliative continuous sedation is used	For what situations do you see a role for palliative continuous sedation to be used?
Clinical approach to palliative continuous sedation	What is your clinical approach to continuous sedation? Which medications do you use? Does your program/institution have specific guidelines for palliative continuous sedation? What parameters do you use to decide if you are using the correct amount of sedation? Sedation can be classified as proportional, which may be lighter, and continuous deep sedation, which is deeper. Do you consider these to be distinct? If so, how? How would you characterize a high quality palliative sedation versus a low quality continuous sedation? Do you measure the quality? What do you primarily intend to accomplish with continuous sedation? Have you ever felt that, by providing palliative sedation, you were at least partially intending to shorten life? What do you explore when talking with your team? What do you explore when talking with the family? Does the family ever influence your clinical management? How do you approach the option of continuous sedation in cases of existential distress?
Impact of assisted death and COVID-19	How would you describe the impact that the legalization of medical assistance in dying (assisted death) has had on your practice of palliative continuous sedation (if any)? How would you describe the impact of COVID-19 on your practices (if any)?
Ethics	What challenges or difficulties do you encounter when considering initiating continuous sedation (if any)?

### Data analysis

Focus groups data analysis was conducted using Interpretative Phenomenological Analysis methodology, according to the following steps: (1) data immersion by reading the first case (i.e., the first focus group); (2) developing emergent themes and associate them to participants’ quotes; (3) searching for connections across the emergent themes; (4) repeating steps 1 to 3 with each case; (5) looking for patterns across cases; (6) presenting a general structure of meaning [31]. This inductive method allowed the researchers to go beyond the initial themes and uncover the latent meaning, enabling an interpretative understanding of the data [37]. 

## Results

Participants shared a common understanding of continuous sedation, despite variations in terminology, attitudes, and practices. Continuous sedation was described as: (a) a last resort intervention for patients whose symptoms can no longer be controlled despite trying all available palliative therapy options (see Tables 4 and 5); (b) an intervention provided when the clinical prognosis is less than two weeks; (c) an intervention that focuses on identifying and alleviating refractory and intolerable symptoms to provide comfort to the patient, their family, and the multidisciplinary team; (d) an intervention that induces unconsciousness in the patient until death; (e) a care that aims not to hasten death, but acknowledges the possibility; (f) an intervention that aims to achieve a good death, as perceived by patient, family, and clinicians.

**Table 4:** Number of focus group participants who reported a symptom warranting the use of continuous palliative sedation

Symptom	N (%)
Delirium/agitation	21 (75.0)
Pain	15 (53.6)
Existential distress	13 (46.4)
Dyspnea/respiratory failure	12 (42.9)
Nausea	2 (7.1)
Hiccups	1 (3.6)

**Table 5:** Number of participants who reported using or wanting to use a medication (grouped by pharmacological class)

Medications	N (%)
Benzodiazepines	
• Midazolam • Lorazepam	21 (75) 7 (25)
Phenothiazines	
• Methotrimeprazine	15 (53.6)
Opioids	
• Hydromorphone • Fentanyl	2 (7.1) 6 (21.4)
General anaesthetics	
• Propofol • Ketamine	5 (17.9) 7 (25.0)
Barbiturates	
• Phenobarbital	11 (39.3)
Anticholinergics	
• Scopolamine	6 (21.4)
Antipsychotics	
• Haloperidol	3 (10.7)
Antihistamines	
• Diphenhydramine	2 (7.1)
Nonsteroidal anti-inflammatory drugs	
• Ketorolac	1 (3.6)
Beta blockers	
• Metoprolol	1 (3.6)
Antiemetics	
• Dimenhydrinate	1 (3.6)
Sedatives	
• Dexmedetomidine	1 (3.6)

Through analysis, six themes emerged, capturing the meaning of continuous sedation for the participants: (1) responding to suffering; (2) grappling with uncertainty; (3) adapting care to ensure ongoing quality; (4) grounding clinical practice in ethics; (5) combining medical, relational, and reflexive abilities; (6) offering an alternative to assisted dying. These themes are presented below with supporting participant quotes.

### Responding to suffering

Continuous sedation addresses the suffering of the patient, the family and, to a certain extent, the palliative care team. Participants primarily perceive continuous sedation as a means to ease the suffering of both the patient and their family, who form a unit of care. The practice surrounding the use of continuous sedation thus involves the observation, the recognition, and the assessment of the experiences of patients and their families:

That’s the big question in palliative care: Whose suffering are we treating? The patient or the family? When we decide to treat death rattle, we don’t treat it for the patients, we treat it for the family. In palliative care, we also see that the interest of the patient often passes through the interest of the family and that it is difficult to separate them completely. Palliative sedation must be seen that way. The quality of care we offer to one is inseparable from the quality of care we offer to the other. (P8, physician)

Alleviating the patient’s suffering can help ease the family’s distress. Families often take the initiative to request continuous sedation when they perceive the patient’s suffering as unbearable. Conversely, they sometimes postpone sedation to ensure the patient remains conscious. Therefore, the family’s experience and consent is at the center of clinicians’ evaluation of suffering. Many participants shared how difficult it can be to challenge the family’s perceptions of the patient’s condition and needs, primarily due to their acknowledgment that the family’s knowledge and experience are legitimate:

I’m going to be humble and say that maybe five minutes before I walked into the room, the patient was agitated, and then he calmed down all of a sudden. The family is going to report that to me and then maybe I won’t have seen it, maybe the nurse won’t have seen it and I’m going to have to believe the family and I’m going to adjust the medication because of that. (P6, physician)

Participants described their exposure to human suffering on a daily basis. This affects them and contributes to their own suffering. Introducing continuous sedation into the patient’s care trajectory offers the possibility of a death free from suffering and therefore is a source of comfort:

There’s a great sense of relief. I don’t know if the right word is satisfaction… It’s hard to watch somebody suffer. To be short of breath, be in pain, to be agitated. And continuous palliative sedation is a nice way to go. It’s a good death. (P3, nurse)

Participants evaluated the quality of continuous sedation in terms of the quality of their response to the suffering of the patient, the family, and the care team:

What would I describe as a good death? Families are all comfortable with the decision. The medical resident is comfortable with the decision. The staff are competent. The symptoms are alleviated fairly quickly, comfortably. Everybody spends quality time together. We have a good debriefing. When death does occur, everybody, you know, they’re sad but they’re happy because it was a peaceful death. Versus it was hard to get the symptoms. We tried so many different interventions to alleviate all the different symptoms the patient was experiencing, and it was really rough getting the sedation going. And they suffered, and there was a lot of screaming. It was upsetting other patients because they were in so much agony. It distressed the staff, it distressed everybody. (P13, physician)

Nevertheless, all participants placed the patient at the center of their concerns and established a hierarchy:

Primarily, relief of the patient’s suffering. And secondarily, relief of the family’s and team’s suffering. (P24, nurse)

### Grappling with uncertainty

For all participants, a prognosis of two weeks or less plays a central role in the decision-making process for continuous sedation. This timeframe not only defines the intent of care but also shapes how palliative sedation is explained to patients and families. However, participants expressed an important level of uncertainty regarding prognosis, highlighting the inherent unpredictability of death and the individualized nature of each case:

Usually, I find I’m pretty good at prognostication if it’s kind of within hours, for sure. Within days, you know, it’s kind of hit or miss sometimes, depending on the situation. (P9, physician)

Clinicians’ uncertainty about prognosis manifested as a form of recognition of their professional limitations. However, this uncertainty did not undermine their confidence in their practice. They maintained a strong sense of obligation based on intention – to alleviate suffering without hastening death – rather than focusing on the potential consequences of their actions:

I like the idea that you kind of convince yourself that the initiation of palliative sedation is not going to hasten things for this person that you’ve started it for irretractable symptoms. (P11, physician)

I think within our group, we all have different comfort levels with palliative sedation. We all say the same thing, but it means a little different things to each of us. (P19, nurse)

Clinicians recognized the importance of making an accurate prognosis as a crucial aspect of providing quality continuous sedation, but errors in timing, whether administering continuous sedation too early or too late, can result in a sense of professional and, to some extent, personal failure:

Statistically, we’ll never get it right. Two weeks of life is still legitimate, but a month… If it becomes very long, it’s very exhausting for families and it’s very difficult in terms of philosophy and explaining what’s going on. (P5, nurse)

### Adapting care to ensure its ongoing quality

Participants perceived continuous sedation as more of a process than a single act. They emphasized the need to constantly adapt care to the situation to ensure its ongoing quality. If participants worked with protocols, they expressed the importance of flexibility in order to accommodate the needs of patients and families, and their own comfort zones:

It’s the art and science of medicine, right? You have to use your clinical judgment, but every individual is unique. You have to modify things based on your good assessments, your gut feeling, your experience. You talk to your team members. (P13, physician)

I use palliative sedation guidelines, just as a matter of course. But often, palliative sedation may be something that I see on the horizon, depending on my history with the patient. Things that I’ve seen, past history. (P21, nurse)

The major impact of the COVID-19 pandemic was clinicians’ inability to properly adapt their practice to the patient-family unit. Due to visitation restrictions, family members were either not present, which undermined clinicians’ ability to respond effectively to patients’ suffering and hindered access to continuous sedation, often initiated by the family:

During COVID-19, probably the biggest challenge was associated with patients and families being physically separated. If a patient was incapable, the family members couldn’t see their loved one to participate in discussions around sedation. So, that became a major barrier to patients who had refractory delirium or respiratory failure or pain being able to access continuous sedation. (P2, physician)

Participants working in home care settings highlighted the need for additional adaptation on the part of families who are involved in sedation at home. The challenge for these families is to adjust the patient’s medication according to clinicians’ instructions:

What we’ve done is maybe put in a PCA [patient-controlled analgesia] pump so the family can readjust the dose as we go, on the hours that there’s maybe no nurse available. So, we’re looking into little projects like that to try to help facilitate things for families. And so that we don’t end up waiting too long to readjust the medication dose. It’s always the continuous conversation that we have with families. (P20, physician)

### Grounding clinical practice in ethics

Participants understood continuous sedation as a care based on ethical thinking and communication. They recounted being confronted with ethical dilemmas, knowing their decisions are fraught with consequences (e.g., causing suffering, modifying life span):

Family members have distress of their own, and it’s very understandable. But as a clinician, there’s always this internal struggle. Is the sedation too soon? Does the family understand what this means? Have I explained it in a way that they understand? How much is too much? How little is too little? Am I prolonging suffering? Have I done enough to get to the point where this is a reasonable intervention? This is the stuff that I think, as clinicians, we do lose sleep over, at times. (P21, nurse)

Communication emerged as an essential aspect of continuous sedation practice. While participants demonstrated respect for the experiences of others, they recognized that continuous sedation entails the confrontation of values, which can give rise to conflicts, misunderstandings, or discomfort:

Patient, family culture versus our own beliefs and our own ethics. It becomes almost a little dilemma at times. And we’ve had multiple arguments and issues about that. (P20, physician)

Conflicts stemming from divergent values and clinical judgments within multidisciplinary teams can also arise, potentially leading to a breakdown of trust among colleagues or within the care practice:

The nurses felt like they knew what was the right thing for the patient, but it wasn’t. Physically, it might’ve been the right thing for her, but from a family, emotional perspective. It was probably one of the most difficult situations I’ve been in. Not because of the patient and the family, but you know, that dynamic with my team, who would not listen to me. It didn’t matter what I said. Things really fell apart quite a bit then. (P19, nurse)

Most participants voiced ethical concerns regarding the use of continuous sedation for patients primarily suffering from existential distress:

We do have other patients who really are awake, alert, compos mentis, and just have tremendous existential suffering. The challenging thing in that scenario is that the degree of existential suffering does not always correlate with prognosis. (P2, physician)

When it’s more kind of psychosocial or existential, then decision making can be a little bit more difficult. And perhaps more controversial. Although, I mean, suffering is suffering. Can you really say that physical suffering should take greater priority over psychosocial or existential? (P9, physician)

### Combining medical, relational, and reflexive abilities

Participants seemed to experience continuous sedation as a combination of medical expertise (e.g., selecting medications, establishing prognosis, using tools and guidelines), relational tact (e.g., opening a dialogue, cultivating presence), and reflexive abilities (e.g., ethical questioning, confronting values):

Sometimes, we think we have to use all these fancy measures and we get these quantitative measures. And then you’re saying, you know, the qualitative. Like that, what did people say? What are the stories that they shared, good or bad? And you get that rich understanding of what happened. Much different than a numerical kind of a measurement. (P13, physician)

Combining medical, relational, and reflexive abilities is a way to provide good care that goes beyond medical sedation:

That’s our accompaniment, right? You can have very good sedation, but that will leave people with a somewhat traumatic grief because it is an end of life that was quite traumatic. This is the opposite purpose of continuous sedation, so there is work to be done. (P8, physician)

Isolation measures and visitor restrictions during COVID-19 pandemic had a greater impact on clinicians’ relational and reflexive abilities compared to their medical skills. The quality of continuous sedation was affected more than its quantity, as the clinical understanding became somewhat detached from the realms of affect and communication. This appeared to reduce the ethical and relational scope of continuous sedation during the pandemic:

It’s very hard when a family can’t see their loved one and know what’s going on, to have that kind of conversation with them over the phone. It’s really hard to have people who only know from an intellectual perspective what’s happening with their loved one to understand that maybe we’ve come to that point. (P19, nurse)

### Offering an alternative to assisted death

The legalization of assisted death in Canada has changed the ecosystem of palliative care. It appears that clinicians and patients viewed continuous sedation as an alternative to assisted death. The majority of participants understood that continuous sedation was different from assisted death in terms of prognosis and intention (i.e., to respect the natural rhythm of dying). They reported having the additional responsibility to educate patients, families, and non-expert healthcare professionals about the distinction between continuous sedation and assisted death.

Other participants understood continuous sedation practice in continuity with assisted death, indicating that these interventions are not mutually exclusive. They believed that offering continuous sedation to certain patients is a suitable compromise that upholds the intention to relieve suffering:

Where I find that palliative sedation is most frequently used are patients who would consider medical assistance in dying, but are now probably too late to get the ball rolling for the application process. So, that’s an interesting middle ground I find for patients who have intractable symptoms, who have intolerable symptoms, and don’t want to be conscious anymore, or can’t be conscious anymore because they’re too agitated. We can sedate people up until their medical assistance in dying procedure. (P1, physician)

Participants’ perception of continuous sedation as an alternative to assisted death was part of a “culture of acceptance” of assisted death among patients, families, and healthcare professionals, reflecting a notable shift in medical and societal norms. However, a disparity arises between the intentions of clinicians practicing continuous sedation and the understanding of patients and families who do not necessarily differentiate between continuous sedation and assisted death:

With some patients, it’s really, “Well, so let me get this straight. As far as I’m concerned, both of them are the same. I go to sleep and I die in my sleep. Sedation, you could do it this afternoon, right? I want that one.” And these are for patients who have limited prognoses. So, I think the cultural acceptance, which in turn, influences patients but also our willingness to discuss it and our competency in discussing it. (P2, physician)

Overall, participants acknowledged a positive impact of the legalization of assisted death. The public debate surrounding it has increased awareness about the realities of end-of-life experiences and fostered conversations about death and palliative care, including continuous sedation:

I think that one of the pluses for palliative care that came out of medical assistance in dying is that we’re talking about palliative care more. All of the aspects of palliative care, so people can make a more informed decision. (P24, nurse)

## Discussion

### Main findings

Our findings show that continuous sedation practice is centered around addressing and assessing the suffering experienced by patients, families, and clinicians. According to the idea that individual experiences are intertwined, two sources of distress justify continuous sedation: that of the patient, resulting from physical, psychological, and/or existential suffering, and that of the family and clinicians, resulting from witnessing the patient’s suffering. Participants perceive continuous sedation as a form of quality care when they are able to effectively respond to and alleviate suffering. This entails recognizing, understanding, adapting to, and mitigating the patient’s suffering. In line with previous research, this study shows that some clinicians prioritize the patient’s suffering as a critical factor, even above considerations of life expectancy alone [7]. 

Our findings highlight that the suffering of families and clinicians is not peripheral but rather central to continuous sedation practice. On one hand, the family can experience suffering as they witness their loved one’s suffering on a daily basis. Clinicians acknowledge this suffering and strive to alleviate it by providing sedation to the patient. On the other hand, clinicians themselves experience suffering due to their continuous exposure to suffering and death, as well as the challenges they face in aligning their care with their own values. [7, 8, 15, 18, 21, 25] Thus, palliative care clinicians adopt strategies to mitigate workplace suffering and share the moral burden, such as supporting each other and deliberating [38]. This suggests that continuous sedation practice is undermined by inadequate care of the patient’s family and insufficient recognition of the emotional and moral experiences of staff members [11, 39]. 

This dynamic understanding of suffering among palliative care clinicians is consistent with the abilities they wish to develop. Our study provides a description of continuous sedation practice as a combination of three abilities: medical expertise, relational tact, and reflexivity. It appears essential for clinicians to support objective monitoring tools with subjective observations and collective discussions [13]. Clinical evaluation should be supported by a process of exchanging, sharing, interpreting, and confronting perceptions. [17, 40, 41] Going beyond medical technique allows clinicians to provide holistic care and better adapt to complex symptoms and complex human components.

Findings also support the idea that palliative care clinicians encounter ethical concerns when practicing continuous sedation. [16, 18, 24, 25, 40] First, our results confirm that clinicians have difficulty making a prognosis although they know it is a selection criterion for continuous sedation [7, 21, 38]. Continuous sedation practice calls into question the death of patients as “natural” [42]. According to our study, palliative care clinicians experience the uncertainty related to prognosis as professional and personal limits to providing a good and natural death. However, they embrace uncertainty as part of their practice and can tolerate uncertainty by connecting with their primary intention and trusting the nature of their act as morally good. The uncertainty related to prognosis may even keep alive an ethical thinking about the use of continuous sedation, since it requires an acute awareness of the legal framework and a rigorous evaluation of patients and families.

Second, our results confirm that pain, delirium, and dyspnea are commonly accepted indications for continuous sedation, while existential suffering is more controversial [15–17]. Existential distress is perceived by clinicians as a symptom with different issues than pain or delirium, possibly because it is considered outside the medical field, requiring specific ethical, psychological, and spiritual assessments. [11, 16, 43, 44] It is not clear for clinicians how to address existential suffering. This points to the need for closer collaboration with other professionals (e.g., psychologists) and improved training [4, 15]. Empathy with the patient’ suffering or positive attitudes towards sedation may lead clinicians to treat existential suffering using continuous sedation despite the ethical issues it raises [7, 23, 43]. Therefore, continuous sedation practice could depend more on clinicians’ beliefs and tolerance regarding existential suffering than on proper knowledge or clear guidelines [14]. Continuous sedation, which aims to relieve the patient’s total pain [45], cannot be optimal if it does not reach beyond patients’ physical symptoms to address also existential issues [4, 46]. 

In line with previous studies, we also found that many palliative care clinicians perceive the relationship between palliative sedation and assisted death as fluid or interchangeable. [26, 27, 43, 44, 47] Palliative sedation is shaped by patients’ desire to hasten death or explicit demands for assisted death, and clinicians themselves understand continuous sedation as an alternative to assisted death for people who are near to death but cannot consent or do not have access to it. The media coverage of assisted death may also result in increased opportunities for clinicians to engage in discussions about continuous sedation [48]. Far from decreasing, there has been a strong growth in palliative sedation in Canada following the legalization of assisted death [19]. 

### Cultural interpretation of the findings

The technical, medical, relational, and moral dimensions of palliative sedation are linked to its institutional and cultural dimensions. Palliative care clinicians are aware of the context in which they work and make sure to keep in mind the social issues surrounding end-of-life care. Continuous sedation practice reflects factors outside the care setting such as cultural meaning-making around death and dying [49–51]. The way clinicians experience the use of continuous sedation and assisted death is an indication of the importance of achieving a “good death”, which has been characterized by the absence of suffering and agitation and the search for individual control [18, 27, 52]. It appears cultural constructions of “good dying” imbue the experiences of palliative care clinicians, and as a result, influence continuous sedation norms.

### Strengths and limitations of the study

This study provides new phenomenological data on palliative care clinicians’ experience of continuous palliative sedation and enhances understanding of the interplay between continuous palliative sedation and assisted death. This may improve knowledge and clinical practice in the palliative care of patients with far advanced disease.

This study has limitations. The results cannot be generalized to all palliative care clinicians, or to countries where assisted death is not legal. Composed almost exclusively of nurses and physicians, our sample is also limited in terms of interdisciplinarity. In addition, as end-of-life practices are a source of public debate, the focus group topic was sensitive. The data collection could have led to a reluctance to share some experiences and values.

## Conclusions

This study provides novel insights into continuous palliative sedation as a response to the suffering experienced by patients, families, and palliative care clinicians. Additionally, it highlights the central role of family and clinician suffering within the context of continuous palliative sedation practice, rather than considering it as peripheral. This study also enhances understanding of the interplay between continuous palliative sedation and assisted dying, describing how palliative care clinicians educate others about the differences between the two interventions, while also utilizing sedation as an alternative to assisted dying. In addition, this study indicates that palliative care physicians and nurses often struggle to explore, understand, and/or address existential suffering. Therefore, its treatment may depend more on clinicians’ beliefs and tolerance rather than on proper knowledge or guidelines. Its treatment may also depend on the lack of psychological and spiritual care providers in palliative care teams.

## Data Availability

Data generated during the current study are available from the corresponding author upon reasonable request.
